# Telemedical emergency services: central or decentral coordination?

**DOI:** 10.1186/s13561-021-00303-5

**Published:** 2021-02-17

**Authors:** Steffen Fleßa, Rebekka Suess, Julia Kuntosch, Markus Krohn, Bibiana Metelmann, Joachim Paul Hasebrook, Peter Brinkrolf, Klaus Hahnenkamp, Dorothea Kohnen, Camilla Metelmann

**Affiliations:** University of Greifswald, Greifswald, MV Germany

**Keywords:** Centralisation, Emergency medicine, Queuing model, Teleemergency doctor, Telemedicine

## Abstract

**Background and objective:**

Teleemergency doctors support ambulance cars at the emergency site by means of telemedicine. Currently, each district has its own teleemergency doctor office (decentralized solution). This paper analyses the advantages and disadvantages of a centralized solution where several teleemergency doctors work in parallel in one office to support the ambulances in more districts.

**Methods:**

The service of incoming calls from ambulances to the teleemergency doctor office can be modelled as a queuing system. Based on the data of the district of Vorpommern-Greifswald in the Northeast of Germany, we assume that arrivals and services are Markov chains. The model has parallel channels proportionate to the number of teleemergency doctors working simultaneously and the number of calls which one doctor can handle in parallel. We develop a cost function with variable, fixed and step-fixed costs.

**Results:**

For the district of Greifswald, the likelihood that an incoming call has to be put on hold because the teleemergency doctor is already fully occupied is negligible. Centralization of several districts with a higher number of ambulances in one teleemergency doctor office will increase the likelihood of overburdening and require more doctors working simultaneously. The cost of the teleemergency doctor office per ambulance serviced strongly declines with the number of districts cooperating.

**Discussion:**

The calculations indicate that centralization is feasible and cost-effective. Other advantages (e.g. improved quality, higher flexibility) and disadvantages (lack of knowledge of the location and infrastructure) of centralization are discussed.

**Conclusions:**

We recommend centralization of telemedical emergency services. However, the number of districts cooperating in one teleemergency doctor office should not be too high and the distance between the ambulance station and the telemedical station should not be too large.

## Introduction

The German emergency medical service is a two-tiered system of paramedics (1–3 years of training) and emergency physicians (Ziegenfuß, 2007). While paramedics attend all emergencies, not every emergency requires an emergency physician. To increase flexibility of the emergency medical service, paramedics and emergency physicians are transported independently in separate vehicles to the emergency site in the so-called “rendezvous system” [1, 2]. In 2014, an emergency physician was dispatched to 46% of all emergencies. Additionally, in 8.8% of emergencies, paramedics decided after the primary examination to call for help by an emergency physician [3].

The rendezvous system allows flexible allocation of emergency physicians and leads to a faster availability of both paramedics and emergency physicians compared with the stationary system (paramedic and emergency physician are dispatched in the same vehicle). This system clearly provides high quality services, but also involves some disadvantages. Firstly, emergency physicians have to be on call 24/7. Although the area covered by one emergency physician is greater than that of the paramedics, emergency physicians still have to reach the emergency site within minutes, which requires a dense network of emergency physicians. Secondly, emergency physicians are sometimes sent to a patient not requiring their services while other patients would require an emergency physician. This results in unnecessary waiting time; in particular as paramedics are not allowed to administer all drugs (e.g. for analgesia) or perform all interventions [4]. Consequently, there is a need to provide emergency physician services “on call”.

During recent years, teleemergency doctors (TED) have been implemented worldwide and in some districts of Germany in order to solve this challenge [5–7]. TED are emergency physicians working from a teleemergency doctor office that is connected to the emergency site to provide telemedicine. Paramedics can contact the emergency physician via telemedicine to ask for support. The teleemergency doctor receives vital signs (e.g. ECG) in real time, can talk to the paramedic and patient and can observe the patient via camera. Based on this information they can give advice, authorize administration of drugs and supervise interventions [8].

The county of Vorpommern-Greifswald (VG) in North-Eastern Germany has implemented such a teleemergency doctor system in 2017 [9, 10]. VG covers an area of almost 4000 km^2^ with a population of approx. 236,000 inhabitants (60 person/km^2^). Hence, it is a very rural area, with the exception of the town Greifswald with 55,000 inhabitants. At the moment, 6 of 26 ambulances are equipped with telemedical technology (NB: when we refer to “ambulance”, we mean an emergency car equipped with telemedical technology). As of September 2020, approximately 4600 emergencies were supported by the teleemergency doctors in VG. Funded originally by the German Ministry of Education and Research, the entire implementation process was evaluated for a period of 2.5 years. The evaluation showed that a teleemergency doctor system can be successfully implemented in a rural area, that the treatment by TED is safe and that there is a high satisfaction among all involved groups (paramedics, emergency physicians, emergency dispatchers and patients) [11]. However, the costs are high and call for an analysis into how efficiency of the system can be improved [12]. Currently, teleemergency doctors are mainly waiting for emergency calls, i.e., most of the time they are not giving advice but simply waiting for the next emergency requiring their support. This is due to the low population density of VG leading to rather few emergencies. Neighboring districts face the same situation: while one neighboring district already started collaboration and is linked to the Greifswald teleemergency doctor office, other districts might want to implement the teleemergency system as well, but might fear the high costs of running a teleemergency doctor office with low utilization. Consequently, the question arises whether we would centralize the teleemergency doctors, i.e., have one teleemergency doctor office for several districts instead of one office in each district.

While centralization seems efficient, it increases the likelihood of the teleemergency doctor being consulted by two or more ambulances at the same time. Treatment of multiple patients simultaneously increases the mental workload of the teleemergency doctor. Consequently, the workload of working with multiple ambulances at the same time has to be analyzed.

This paper evaluates the pros and cons of centralized and decentralized teleemergency doctor offices in order to support policymakers of emergency care systems to decide on the implementation strategy of a teleemergency doctor system in their districts, which combines qualitative and quantitative dimensions of the decision. We want to prove that the centralization decision can be based on rigid methodology and that a thorough analysis of hard and soft facts can contribute very practically to the policy- and decision-making process. In the next section, we develop a queuing model in order to calculate the number of teleemergency doctors required and the respective costs of the teleemergency doctor services as a function of the number of teleemergency cars serviced. Afterwards we demonstrate the results, i.e., the number of teleemergency doctors required as a function of the number of teleemergency cars and their costs. The paper closes with a discussion focusing on the advantages and disadvantages of central and decentralized solutions as guidance for policymakers.

## Methodology

### Teleemergency doctor office

The teleemergency doctor works in an office with a computer system showing all relevant data sent from the emergency site on four screens. Usually, the teleemergency doctor can handle several calls at a time in parallel, but certainly, there is a limit to that number. If the number of calls in service exceeds that limit, the next calls will have to wait until at least one emergency under service is finished. Usually, every call is picked and answered immediately, but if the teleemergency doctor is already overburdened, the incoming call will be put on hold until full attention and service can be provided. From a medical perspective, the main question is: “What is the likelihood that an incoming emergency call from the paramedic cannot be serviced immediately, i.e., how high is the service availability?” From an economic perspective, the main question is: “How many teleemergency doctors do have to work in parallel in a teleemergency doctor office and how many districts can be centralized in one teleemergency doctor office assuming that a predefined service availability has to be maintained?”

**Fig. 1:**
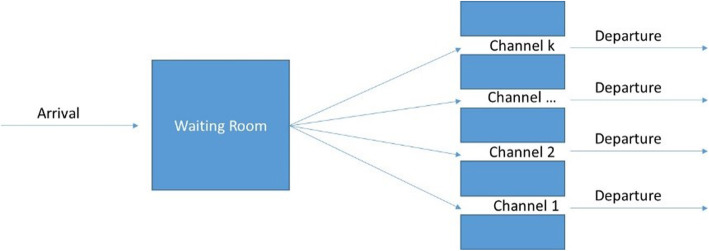
Queuing model

In order to answer these questions, we developed a queuing model [13, 14]. Figure 1 shows a simple queuing model. The customers (here: calls from paramedics) arrive in the waiting system and are serviced. The system has k parallel service channels, i.e., if at least one service channel is free, then the customer does not have to wait. The incoming call only has to wait if all channels are occupied. The main characteristics of the system are:

- Arrivals: How many calls come in a certain period of time and according to which distribution? It has been shown that most arrivals follow a Markov process and are Poisson distributed [15].
- Channels: The number of parallel channels (k) determines how many clients can be serviced at the same time.
- Service: How long does a service take and how is this service time distributed? It has been shown that most service times are negatively exponentially distributed [15].
- Capacity: Some systems have a limited capacity of the waiting room so that incoming customers are rejected if the waiting room is full. Similarly, patients can depart if the queue or waiting time exceeds a certain figure.
- Priority: It is assumed that first customers are serviced first (first in – first out, FIFO), i.e., medical priorities are not considered.

The service of the teleemergency doctor office can be expressed as a queuing model:

- Arrivals: Calls from the paramedics reach the teleemergency doctor office with a rate of λ (calls per hour). It is assumed that the number of calls is Poisson distributed, i.e., the arrival is a Markov process.
- Channels: Each teleemergency doctor can service a maximum of m emergencies at a time. Assuming that n teleemergency doctors work in one teleemergency doctor office in parallel, k = m*n channels are available.
- Service: A contact between the paramedic and the teleemergency doctor takes 60/μ minutes where μ denotes the service rate, i.e., number of services possible per hour. As we assume that the service time of customer *i* does not depend on the service time of customer j, we can assume a Markov process with a negative exponential distribution [16].
- Capacity: We assume unlimited capacity, as the paramedics are employees of the same emergency system as the teleemergency doctors, i.e., they will wait until the call is picked up.
- Priority: We assume FIFO. In reality, there are some calls with an extraordinary priority, but it is extremely rare that it is required to change the priority of handling calls.

### Model: M/M/k; ∞, FIFO

The model assumes a distribution of arrivals according to Poisson distribution (rate λ), a distribution of the length of service according to an exponential distribution (rate μ). The probability density function of the exponential distribution is given as *f*(*x*) = *αe*^−*αx*^ with $E(x)=\frac{1}{\alpha }$ and $\sigma (x)=\frac{1}{\alpha }$, i.e., the coefficient of variation is 1. Furthermore, we assume k = m*n parallel channels, no limitation of waiting room and a FIFO priority. We define the traffic density as $\rho =\frac{\lambda }{\mu }$ and the probability that *i* calls are in the system as *w*_*i*_. The basic formulae are given as [17]:

$$ {w}_0=\frac{1}{\sum_{i=0}^{k-1}\frac{1}{i!}\bullet {\rho}^i+\frac{\rho^k}{k!\bullet \left(1-\frac{\rho }{k}\right)}}=\frac{1}{\sum_{i=0}^{mn-1}\frac{1}{i!}\bullet {\rho}^i+\frac{\rho^{mn}}{(mn)!\bullet \left(1-\frac{\rho }{mn}\right)}} $$

$$ {w}_i=\frac{1}{i!}{\rho}^i{w}_0\ \mathrm{for}\ \mathrm{i}<m\ast n $$

$$ {w}_i=\frac{1}{k!\bullet {k}^{\left(n-i\right)}}{\rho}^i{w}_0=\frac{1}{\left(m\bullet n\right)!\bullet {\left(m\bullet n\right)}^{\left(n-i\right)}}{\rho}^i{w}_0\ \mathrm{for}\ \mathrm{i}\ge \mathrm{m}\ast \mathrm{n} $$

where

*ρ* traffic density

*λ* rate of arrivals [arriving calls per hour]

*μ* rate of service [completed calls per hour]

*k* number of parallel channels

*m* maximum number of calls one teleemergency doctor can service in parallel

*n* number of teleemergency doctors in the teleemergency doctor office

*σ* standard deviation of number of completed calls per hour

*w*_*i*_ probability that i calls are in the system

Based on the probabilities of i calls in the system (*w*_*i*_)*,* we calculated the probability that a call cannot be serviced instantly as

$$ \mathrm{P}\left(\mathrm{i}>k\right)=1-\mathrm{P}\left(\mathrm{i}\le k\right)=1-{\sum}_0^k{w}_i $$

and define this situation as “overburdening”.

### Cost function

The cost of the teleemergency doctor services are determined by the cost of running a teleemergency doctor office and the cost of equipping the ambulances with telemedicine. The latter will be ignored as the respective costs occur whether the service is provided centrally or decentrally.

The analysis of the costs of TED services could be easily separated from the cost of running the original functions of an emergency service because the two functions and offices are separated in the district of Vorpommern-Greifswald. During the project implementation, the standard function of emergency care was separated from the TED project not only legally, but also in terms of accounting and location. Until today, the TED service is in a building of the university hospital and staffed mainly by personnel of the university, while the standard emergency service is under the district commissioner. Consequently, the calculation of costs for TED did not require any allocation of costs to different cost centers. We calculated “full sustainable costs”, i.e., no subsidy was given from the hospital to the TED service and we assumed that the service would continue to exist for a long time, asking always “What costs will occur if we continue this service?”

The cost components of the office are fixed, jump-fixed and variable. The fixed costs do not depend on the number of emergencies or the number of teleemergency doctors working in an office, i.e., mainly the position of a coordinator, office space for administration and obligatory quality management. The jump-fixed costs depend on the number of teleemergency doctors working in one office in parallel, including depreciation of the technical equipment and network as well as the cost of the license for the software. The main component is the salary of the staff working at this office (24 h/day, 365 days/year). The variable costs per emergency will be identical between centralized and decentralized solutions and will therefore not be considered.

We follow the cost function provided by Suess & Fleßa [18].

$$ {C}_d=n\bullet \left({C}_f+v\right) $$

$$ {C}_c={C}_f+e\bullet v $$

where

*C*_*d*_ total cost of decentralized solution for n districts

*C*_*c*_ total cost of centralized solution for n districts

*n* number of districts

C_f_ fixed cost of establishing a teleemergency doctor office

*v* variable cost of running one teleemergency doctor for 24/365

*e* number of teleemergency doctors working in parallel in one office, e = f(n)

The cost functions assume that fixed and jump-fixed costs are identical for the centralized and decentralized TED offices. For salaries, training of staff, provision of network and depreciation of equipment, it is obvious that costs increase with the number of teleemergency doctors working in parallel in one office (e). There is no reason to assume that these items will be more or less expensive if we have one or more TED working in parallel. The fixed costs like rent, support, quality management as well as administration and supervision might jump as well if a critical number of teleemergency doctors working in parallel in one office is reached. However, the model assumes that this number is not achieved under realistic conditions.

The number of teleemergency doctors working simultaneously in one office depends on the number of telemedically equipped ambulances serviced per district. The original setup in Vorpommern-Greifswald is 6 of 26 ambulances, but the function also allows calculating the variable *e* if all ambulances are equipped with telemedical technology and are included into the teleemergency doctor services.

### Data

We used the documentation of the telemedicine office of the district of Vorpommern-Greifswald in North-Eastern Germany to estimate the rates and distribution. Digital data from April 1, 2018 to February 29, 2020 was available. Distribution fitting was done with Stat-Fit3. The maximum number of parallel calls serviced by one teleemergency doctor was estimated by interviewing the respective personnel.

The cost estimates were taken from the district of Vorpommern-Greifswald [18] shown in Table 1. Thus, we receive:

$$ {C}_d=n\bullet 696949 $$

$$ {C}_c=148876+e\bullet 548073 $$

**Table 1:** Costs of teleemergency doctor services (p.a.). Source: Calculations based on [18]

Category	Subcategory	Cost [€]	Total [€]	Variable
Fixed costs	Rent	7200		
	Support	3116		
	Quality management	27,560		
	Supervision and administration	111,000	148,876	C_f_
Jump-fixed costs	Salary	508,144		
	Training	6924		
	Network	20,797		
	Equipment (depreciation)	12,208	548,073	*v*
Total costs per office			696,949	C_f_ *+ v*

## Results

### Descriptive statistics

From April 1, 2018 to February 29, 2020, the teleemergency doctor office serviced 3019 emergencies, e.g. 4.14 emergency calls per day. 253 emergencies required one minute or less than one minute and were excluded from the service time analysis resulting in 3.96 emergencies per day. Furthermore, emergencies with a total duration of more than 4 h were excluded (three emergencies). For the remaining 2763 values, the service time ranged from 1:03 min to 3:32:19 h, averaging 24:37 min. Standard deviation was 928.19 s.

**Fig. 2:**
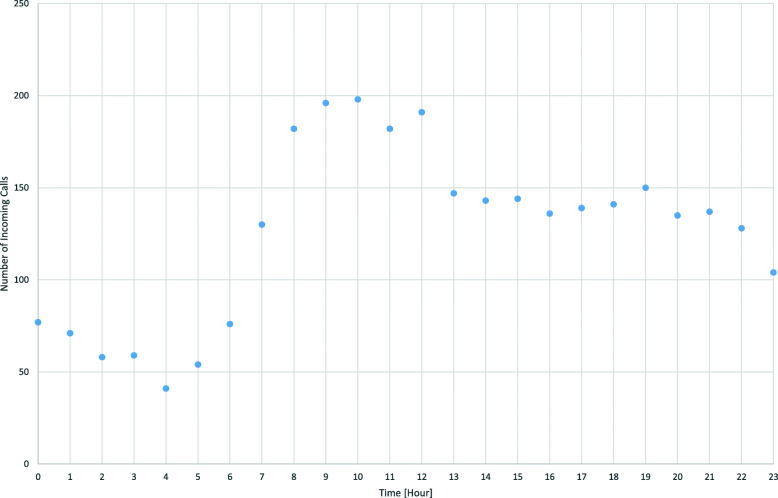
Teleemergency doctor calls per hour

Occasionally, one teleemergency doctor had to service two emergencies at a time. The teleemergency doctors interviewed stated that two would also be an appropriate maximum number of emergencies to be serviced in parallel. Figure 2 shows the number of calls during this period starting in a certain hour (e.g. 130 calls from 7 to 8 a.m.). It is obvious that the number of incoming calls is highest between 8 a.m. and 1 p.m. with an absolute peak between 10.00 a.m. and 11.00 a.m.

**Fig. 3:**
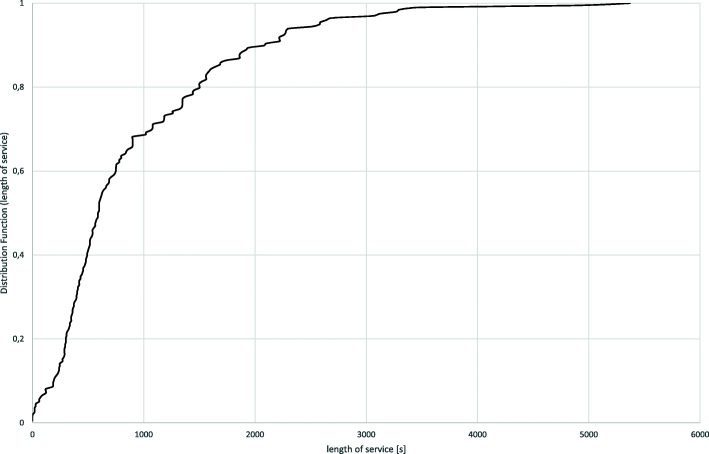
Distribution function of length of service

For the entire period, the rate of arrivals is λ = 0.18; for the peak period it is λ = 0.27. The service rate is μ = 2.57 for all 3019 calls or μ = 2.36 for 2823 values ≥60 s. Distribution fitting shows that the assumption of a Markov process of arrival cannot be rejected, i.e., it can be assumed that the number of arrivals per hour follows a Poisson distribution. For the service process, the exponential distribution should be theoretically appropriate [16, 17], but the respective tests of CHI-2 and Kolmogorov-Smirnov do not lead to a significant result. However, Fig. 3 shows that a negative exponential distribution is at least acceptable and can be used as approximation.

### Actual data from the district Vorpommern-Greifswald

Consequently, the following calculations for the existing teleemergency doctor office in Vorpommern-Greifswald are based on an M/M/2*m; ∞, FIFO model with λ = 0.27 and μ = 2.57, i.e., we concentrate on the peak hours of arrival to calculate the arrival rates and determine the service time under the assumption that it is independent from the time of arrival. With these parameters we calculate w_0_ = 89.97%, i.e., the likelihood that the teleemergency doctor has no service provision in almost 90% of the time. The probability that a call will have to wait for service because the teleemergency doctor is already fully occupied with two emergencies is 0.03%, i.e., in reality, the teleemergency doctor is rarely overburdened. Even strong increases of the arrival rates will not make a major difference. In order to receive a rate of calls having to wait of 10%, the arrival rate has to be 2.093, i.e., 7.7 times as high as shown in the empirical data from this district. Assuming that the need for tele-emergency doctor services is unlikely to be that high, this calls for centralization and cooperation with other districts.

Currently, six of 26 ambulances are equipped with telemedical technology. The selection was based on an analysis of the frequency of emergency physician services and distances, i.e., we concentrated on very rural areas of the district. Our analysis shows that even if the district of VG had equipped all 26 ambulances with telemedicine technology (λ = 1.17), the likelihood that an incoming call has to wait is approx. 2.01%, i.e., even a very big and rural district can work with one teleemergency doctor.

### Centralization

**Fig. 4:**
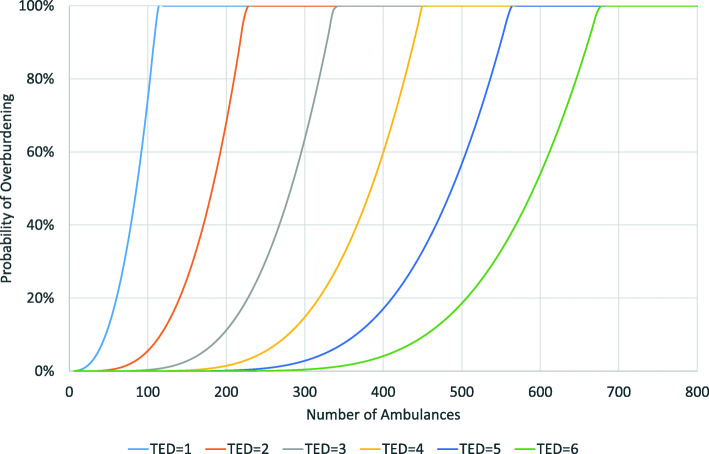
Probability of overburdening as a function of the number of ambulances and the number of teleemergency doctors

We assume that all districts follow the same distribution of arrivals with identical rates (λ). Similarly, we assume that the service time of all TEDs follows the same distribution with identical rates (μ) regardless of the district and the degree of centralization. Under these conditions we calculate the probability that a teleemergency doctor office is overburdened, i.e., that an incoming call from a paramedic has to wait. Figure 4 shows the results.

As stated before, the probability that the system is overburdened is 0.03% if one TED is responsible for one district with six telemedical ambulances (situation in Vorpommern-Greifswald). The probability increases to 0.22% if telemedical emergency services of 12 ambulances (two districts like Vorpommern-Greifswald) are centralized in one office, 19.81% for 60 ambulances (10 districts) and 56.96% for 90 ambulances (15 districts). With 114 ambulances (19 districts) the likelihood is 100%, i.e., the system fails to cover all incoming calls. If we deploy two TEDs in one office in parallel, the respective likelihoods are smaller at a given number of districts. As the figure shows, two TEDs can cover up to 228 ambulances (38 districts like Vorpommern-Greifswald), but then almost all calls have to wait. The figure also shows the probabilities for three to six TEDs in parallel clearly indicating that the number of TEDs strongly determines the likelihood of overburdening.

**Fig. 5:**
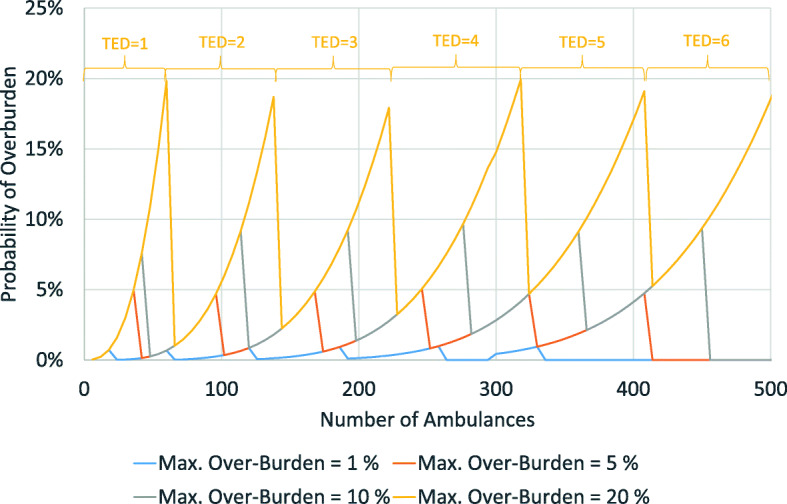
Probability of overburden at different maximum probabilities. Note: the required number of TEDs is only indicated for a maximum probability of overburdening of 20%

Assuming a certain maximum probability of overburdening can also indicate the number of required TEDs. Figure 4 shows that a maximum probability of overburdening of 20% can be reached by one TED with 90 ambulances (15 districts), by two TEDs with 210 ambulances (35 districts), by three TEDs with 330 ambulances (55 districts) and by four TEDs with 480 ambulances (80 districts). Based on this analysis we can calculate the optimum number of TEDs depending on the maximum probability of overburdening. Figure 5 shows the results for a maximum probability of overburdening of 5, 10, and 20%.

If we assume a maximum probability of overburdening of 5%, one TED can handle up to 36, two TEDs up to 96, three TEDs up to 162, four TEDs up to 240, five TEDs up to 324 and 6 TEDs up to 408 ambulances (6, 16, 27, 40, 54 and 68 ambulance districts like Vorpommern-Greifswald respectively). If we assume a maximum probability of overburdening of 10%, the respective figures for ambulances are 42, 114, 192, 270, 360 and 456. For a probability of 20%, it is 60, 138, 222, 318, 408 and 504. If we want, for instance, to centralize 40 districts with a total of 240 telemedical ambulances and have a maximum likelihood that an incoming call has to wait of 10%, we will need 4 TEDs.

### Cost function

**Fig. 6:**
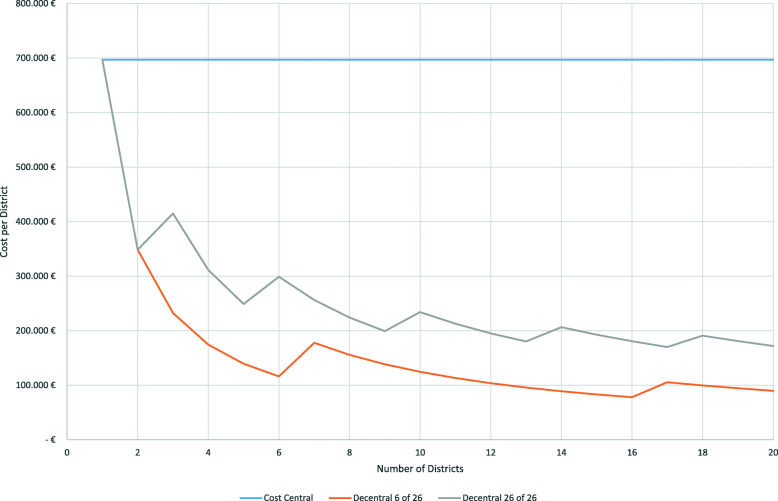
Cost functions

Figure 6 shows the unit cost functions for a centralized system and for two different versions of decentralized systems. The red curve illustrates the current situation in Vorpommern-Greifswald with 6 out of 26 telemedical ambulances (“Decentral 6 of 26”). The grey curve shows the unit cost function if all ambulances are included in the telemedical emergency doctor services (“Decentral 26 of 26”).

For the following analyses a maximum likelihood of overburdening the system of 5% is assumed. If each district runs its own tele-emergency doctor office, the annual cost are about 696,900 € per district. The costs per district are identical irrespective of whether only 6 of 26 (23%) or all ambulances are linked to the office. This is due to the fact that one tele-medicine doctor can service up to 36 ambulances without increasing the likelihood of delaying a call above 5%.

If we centralize and combine several districts in one office, the unit cost per district strongly declines. A sharp cost decrease occurs, if the number of TEDs working simultaneously in a centralized station increases. If we assume that each district runs 26 ambulances, 20 districts will require 6 emergency doctors working simultaneously. The annual costs per district are approx. €171,900. This equals 24.6% of the costs of a decentralized solution with the same maximum likelihood of putting an incoming call on hold. However, many districts are not as big as Vorpommern-Greifswald, have fewer ambulances and might not equip all cars with the respective technology. Hence, the financial advantages of centralization will even be higher. Assuming that 23% (6 of 26) of ambulances are included in the teleemergency doctor service, 60 districts will require 6 emergency physicians working simultaneously in one central office. The annual cost per district will be approx. €57,300, i.e., 8.2% of the decentralized solution.

## Discussion

### Advantages of a centralized system

The calculations presented in this paper clearly highlight that there are strong advantages to centralization of the services of teleemergency doctors in one office for several districts. In particular, the costs for the entire system and for each district are much lower if we centralize as compared to a decentralized solution. This is mainly due to the fact that the utilization of the TED’s working place is extremely low if one TED is responsible for one district. Even if all 26 paramedic ambulances in the district of Vorpommern-Greifswald were equipped with telemedical technology, the likelihood of being put on hold is approx. 0.5%. Furthermore, even for the district with the highest number of ambulances in the state of Mecklenburg-Western Pomerania (Mecklenburg Lake-District) with 40 cars, one TED can handle all of them and the likelihood that an incoming call has to wait is less than 2%. Setting up the respective office is expensive, but the most important cost component is the salary of the teleemergency doctors. Providing the service 24 h per day for 365 days per year requires at least five doctors (each working 230 days per year á 8 h/day). As a decentralized solution leaves this valuable resource mainly idle waiting for the next call, strongly underlines the needs for centralization. At the same time, the higher workload per TED will lead to fast learning and might decrease the time required per patient (economies of scale).

At the same time, the number of TEDs in a centralized solution might even be higher than indicated in this calculation. We used data from the peak hours of the day (see Fig. 2) in order to cover the “worst case”. If we have one TED office per district, the number of TEDs must be one throughout the day and the week. If we centralize, the number of TEDs could be lower than the calculated number of TEDs for night shifts and weekends. This will result in lower costs of the centralized solution and underline the advantages of centralization.

Furthermore, the quality of teleemergency doctor services is likely to increase after centralization. If more than one TED works in the office, they can support each other in case of an emergency or a very difficult case. It might also be possible to have sub-specializations if more TEDs are deployed in parallel, such as a TED pediatrician. In addition, education of TED might be more efficient if provided for a greater number of staff in one office.

Centralization might also lead to a situation in which managing the teleemergency doctor office and the entire rescue system might be easier and more efficient. Physicians are a scarce resource in Germany and emergency doctors with the special training in emergency medicine and telemedicine are particularly scarce [19]. Therefore, the high number of TEDs required in the decentralized system might simply not be available on the market regardless of costs. Thus, reducing the number of TEDs by centralization might relax the staffing problem. Furthermore, many highly qualified physicians want to work in cities, not in rural districts [19]. Centralization might allow TEDs to live and work in the cities but still service emergencies in rural places. Beyond that, having more TEDs in parallel in a centralized office might support the management by facilitating more flexibility. If one TED unexpectedly falls sick, the others of the same shift can cover the workload. It might also be easier to plan night and weekend shifts and provide full service availability during holidays.

### Advantages of a decentralized system

On the other hand, a decentralized solution has a number of advantages as well [20]. Most convincing, a decentralized solution ensures that TEDs have local knowledge. They know the location of the emergency, the road system, the hospital infrastructure and the paramedics. Thus, they can make decisions based on evidence incorporating all knowledge of the local situation. One might argue that the paramedics can support the TEDs with that knowledge, but this will require more time and involves the risk of losing information. Thus, a decentralized system might lead to a better outcome.

Another disadvantage of centralization might be increased stress of TEDs. The likelihood of servicing more than one call at a time increases for the centralized solution while the idle time between calls decreases. Consequently, the workload for TEDs is higher for the centralized solution. This might lead to stress, high fluctuation and difficulty attracting staff. However, long times between services might also lead to boredom and make the job as a TED unattractive. Currently, it cannot be decided which of the arguments will be stronger.

Additionally, a decentralized system has the advantage that a complete breakdown of the system will only affect one district. The existential risk (e.g. fire, longer electricity cuts, strikes) is low, but significantly higher than zero. Thus, central systems are more prone to existential risks. One might overcome this hazard by providing reserve capacities, but this will induce higher costs of the centralized solution.

The management of a decentralized system might also be more efficient as the TEDs and paramedics know each other. They develop trust in each other, thereby speeding up the service process [21]. A TED might even distinguish his or her decisions and advice if he or she knows the ability of the individual paramedic. The flexibility might also increase if TEDs know the local situation as they can react to the local situation due to their familiarity with the current conditions (e.g. detours, weather, events).

Finally, Germany is a federal republic and emergency systems are the responsibility of the federal states. This means that Germany has 16 different laws of emergency services with deviating regulations on rights and obligations of paramedics, peculiarities of the rescue chain (e.g. use of helicopter services) and limits of service times. It is difficult for a strongly centralized teleemergency doctor office to know all the regulations for different states.

Without a doubt, some of the disadvantages of the centralized system can be healed by providing professional software support for the central TEDs, incl. Detailed online knowledge of weather conditions, regulations, paramedics, etc. Keeping this information updated is quite an effort not included in the calculations given above.

The patients or relatives might prefer local solutions. Once the paramedic reaches the patient at the site of emergency, they explain to the patient or relatives that they will now contact the teleemergency doctor. For many people – in particular in rural areas in remote districts – it might make a difference whether the emergency paramedic explains that the TED is situated in the administrative center of this particular district or hundreds of kilometers away in a major city.

Table 2 summarizes the advantages and disadvantages of centralization. It is clear that covering all districts of Germany with independent teleemergency doctor offices is a waste of resources and that having only one or few major offices for all districts in Germany might not be feasible due to the need to know the local situation, the personnel and laws. However, centralization and decentralization are not a binary variable. Instead, we propose a centralization of teleemergency doctor services per state. Even the small state of Mecklenburg-Western Pomerania with eight districts, 1.6 million inhabitants, and a population density of 69 people per km^2^ will benefit a lot from a centralized system. Instead of having costs of approx. €696,900 per district, centralizing eight districts in one office will lead to costs per district of approx. €87,100 (assuming that 23% of all ambulances are equipped with telemedical technology) or some €224,100 (assuming that all ambulances are equipped with telemedical technology). At the same time, the laws and regulations are identical for all districts. It might even be possible to get to know each other in one state. For other states of Germany (e.g. Bavaria with 71 districts and 25 county boroughs), it might be worthwhile to have 2–3 offices in different parts of the state in order to ensure that familiarity is not lost.

**Table 2:** Advantages and disadvantages of centralization

Criteria	Sub-criteria	Advantage	Disadvantage
Cost	Equipment and setup	Higher utilization leads to lower cost per service unit	
	Personnel	Higher utilization leads to lower cost per service unit	
	Economies of scope and scale	Learning effects lead to lower cost per service unit	
Quality	Knowledge of location		Detailed knowledge of location, infrastructure and peculiarities is not possible
	Safety	TEDs can support each other in complex situations	
	Learning effects	Higher routine, sub-specialization of TEDs	
	Existential risks		Collapse of central system means breakdown for all districts
Management	Cohesion of the team		Decentralized system induces stronger trust between paramedics and TEDs as well as better knowledge of the strengths and weaknesses of the respective partner
	Staffing	Lower number of TEDs required	Stress for TEDs
	Further education	Economies of scale	
	Flexibility	Higher flexibility for staffing (e.g. night, weekend, leave, unexpected sickness)	Inflexible reaction to changes of local circumstances
Structures	Laws and regulations		Different emergency laws between German states limit centralization
Patients	Trust of patients		Patients might trust a TED more knowing that they are located in a nearby city

### Limitations

This analysis is subject to a number of limitations. First, our analysis is based on the specific situation in the district of Vorpommern-Greifswald. Our data represents the empirical facts from this district and might have to be adjusted for other districts. For instance, office rent and salaries are lower in the Northeast of Germany than in other districts.

Secondly, the queuing model has a number of assumptions which might be challenged. The assumption that arrivals and services are Markov processes is well founded in theory, but we could only prove it for arrivals, not for services. Another distribution of departures might change the respective calculations slightly. However, the financial advantage of centralization demonstrated with this queuing model is so strong that another model might lead to slightly different numerical results without any impact on the evidence provided.

Thirdly, our rate of arrival was based on the peak-period from 10.00 a.m. to 11.00 a.m. with a rate of arrivals of λ = 0.27. The logic behind this assumption is that the main objective of our calculation is to avoid overburdening the system. By assuming the maximum arrival rate as the model parameter, we ensure that the risk of letting a call wait is lower for all other periods. However, this might underestimate the advantages of centralization at other times of the day, i.e., centralization might be more economical in times where occupancy of the TEDs is even lower.

Fourthly, our model assumes that fixed costs (e.g. rent, quality management, supervision) do not depend on the number of TEDs working in parallel. This is not true for very high numbers of TEDs. As we recommend establishing one central TED office in the state of Mecklenburg- Western Pomerania and 2–3 in states with more inhabitants and emergency cases, this critical point where fixed costs jump will not be reached, but the general problem that what we call “fixed” might not be “totally fixed” should be kept in mind. Otherwise, we might overestimate the advantages of centralization.

Fifth, one major assumption of the model is that the traditional emergency service (dispatchment, fieldwork and office support) are separate from the teleemergency doctor service. This was the situation in Vorpommern-Greifswald during the implementation stage of the project. However, once the teleemergency doctor services have been established as a standard, dispatchment and TEDs could be combined in one office and processes could be interrelated. This would lead to synergies with an impact on costs. However, this future event could not be reflected in this paper.

## Conclusions

Even with these limitations, we can conclude that there is clear evidence that centralization is economically wise. Several authors have shown that teleemergency systems are effective from a medical perspective [22–24] as well as efficient from an economic [25, 26] perspective. Our analysis strongly underlines that the efficiency strongly increases if the teleemergency doctor offices are centralized, but at least under German conditions of federalism, centralization is limited. The first step in this direction is to determine the acceptable probability of overburdening, i.e., the likelihood that an incoming call from a paramedic will not be serviced immediately. Politicians should determine whether a rate of 5, 10% or 20% is acceptable. At the same time, it must be decided whether all ambulances should be equipped with telemedical technology or whether this innovation should be limited to very remote areas only. Based on these decisions, we can determine the number of TEDs working simultaneously. Here we should also analyze whether the rate of arrivals (λ) is indeed identical for all districts and – if not – adjust the calculations accordingly. The location of this office should be determined as well, but this will require negotiations between the district governors. Thus, it will still take some time until the centralized TED system is fully implemented in the entire state, but it is definitely worthwhile starting the process now in order to prevent each district from building its own office with all the disadvantages shown in this paper. The experiences from Vorpommern-Greifswald could serve as an innovation seedling for teleemergency care in Germany.

## Data Availability

All data used for this article was freely accessible within the project.

## Abbreviations

e.g.: Exempli gratia (for example)
ECG: Electrocardiogram
EMS: Emergency medical service
FIFO: First in – first out
i.e.: Id est. (that means)
NB: Nota bene (note well)
p.a.: Per annum (per year)
TED: Teleemergency doctors
VG: Vorpommern-Greifswald
